# Improvement of Electronic Health Record Integrated Transition Planning Tools in Primary Care

**DOI:** 10.1097/pq9.0000000000000282

**Published:** 2020-05-18

**Authors:** Jack Rusley, Kathy Tomaszewski, Julia Kim, Larnce Robinson, Kadi-Ann Rose, Caroline Aronin, Matthew Molloy, Renata Arrington-Sanders

**Affiliations:** From the Division of General Pediatrics and Adolescent Medicine, Department of Pediatrics, Johns Hopkins University School of Medicine, Baltimore, Md.

## Abstract

**Introduction:**

The transition from pediatric to adult care is under-studied in primary care. Electronic health record-integrated transition planning tools (ETPTs) can facilitate the transition. We tested whether ETPTs and clinician reminders increase the frequency of transition discussions during adolescent well-visits.

**Methods:**

In an academic adolescent medicine primary care practice serving a predominantly African American, Medicaid-insured population, we developed 4 ETPTs—readiness assessment, plan template, information handout, and diagnosis code. We used Plan-Do-Study-Act quality improvement cycles to implement ETPTs and measure outcomes. Each cycle added a new layer of clinician support: (1) ETPT training, (2) visual reminders, (3) incentives, and (4) daily reminders. The primary outcome was the proportion of well-visits in which “any ETPT use” occurred. We collected data via chart review and used run charts and regression analyses with multiple comparisons to detect differences between cycles. Clinicians-provided feedback was elicited.

**Results:**

Any ETPT use increased from 0% to 45% between baseline and cycle 4. The odds of any ETPT use was ten times larger in cycle 4 compared to cycle 1 (odds ratio 10.09, 95% confidence interval 2.29–44.44, *P* = 0.002) and 22 times larger in cycle 4 than cycle 2 (odds ratio 21.99, 95% confidence interval 3.96–122.00, *P* < 0.001). Clinicians identified time constraints and lack of sociocultural relevance as barriers to uptake.

**Conclusions:**

Daily reminders combined with training and visual reminders were effective in increasing the use of ETPTs in primary care. Future interventions should adapt existing transition tools to the needs of target populations and create regular reminders to facilitate uptake.

## Abstract 2

Supplemental Digital Content is available in the text.

## INTRODUCTION

Transition to adult-oriented health care for adolescents and young adults (AYAs) is often complicated by gaps in care, deterioration of health, and unmet health needs.^1–3^ Challenges to successful transition include lack of routine discussions about transition between health care clinicians, youth, and families; failure to assess transition readiness; and lack of tools to facilitate transition discussions.^4^ Ideally, transition preparation is centered in the primary care medical home, starts in early adolescence, and includes youth with and without special health care needs.^5^ However, most studies have occurred in pediatric sub-specialty settings in a single chronic health condition, such as cystic fibrosis.^6–9^ A recent systematic review of 1,888 transition studies found that only one took place in primary care.^10^ McManus et al conducted a quality improvement (QI) study. They demonstrated that transition planning tools could be incorporated into primary care settings, but lack of integration with electronic health record (EHR) systems limited the sustainability of the intervention.^11^ A more recent review found that structured transition interventions—such as those based on the Six Core Elements^12^ developed by Got Transition/Center for Health Care Transition Improvement—often resulted in positive outcomes; however, none of the 43 studies examined included youth without special health care needs.^13^

Few studies have addressed how best to integrate transition planning tools into EHR.^14^ EHR-integrated transition planning tools (ETPTs) have been developed in pediatric sub-specialty settings and are acceptable to youth, families, and clinicians with high clinician uptake.^15,16^ Experts have advocated for the development of ETPTs in primary care settings, and suggest these tools could: (1) improve the consistency and frequency of data collection about transition readiness, (2) facilitate a multi-disciplinary approach to transition planning, and (3) enable evaluation and dissemination of EHR-integrated transition activities.^14^ To our knowledge, there have been no studies on integrating ETPTs into primary care, nor have studies examined transition interventions for youth without special health care needs. Therefore, we created a QI intervention^17^ based on the Six Core Elements using a Plan-Do-Study-Act (PDSA) approach^17,18^ to increase the proportion of ETPT use from 0% to 40% over 8 months in our primary care practice serving AYA with and without special health care needs. We based this target on our prior experience with the uptake of QI interventions.^19^

## METHODS

### Context

The setting was an urban academic ambulatory care center for adolescent medicine located within a pediatric center, serving approximately 1,500 AYAs between the ages of 17–26 who live in East Baltimore, Maryland, with sizable Medicaid-insured (90%), African American (90%), and special health care need^20^ (40%) populations. Our intervention focused on clinicians (n = 40), which included resident physicians on their adolescent medicine rotation (n = 25), medical students on their adolescent medicine elective rotation (n = 3), adolescent medicine fellows (n = 5), a nurse practitioner, and adolescent medicine physician faculty (n = 6). The clinic team also included a nurse-clinical coordinator, a social worker, a psychologist, medical assistants, and nurses. EPIC (Verona, WI) is the institution’s EHR system.

Prior work in our clinic on transition and the barriers to success are as follows. We had developed a transition policy as a paper brochure (**Supplemental Material A, Supplemental Digital Content 1**, http://links.lww.com/PQ9/A179), but clinicians distributed it inconsistently. The Transition Readiness Assessment Questionnaire^21^ was adapted to identify youth with special health care needs and track specific transfer tasks (eg, make an intake appointment with an adult-oriented clinician). However, this paper form (**Supplemental Material B, Supplemental Digital Content 1**, http://links.lww.com/PQ9/A179) was not routinely administered or reviewed at subsequent visits. Before this project, a group of youth and parents in our clinic screened the transition policy and the readiness assessment for clarity and relevance and made minor adjustments.

### Intervention

#### Improvement Team and Theory

**Fig. 1.**
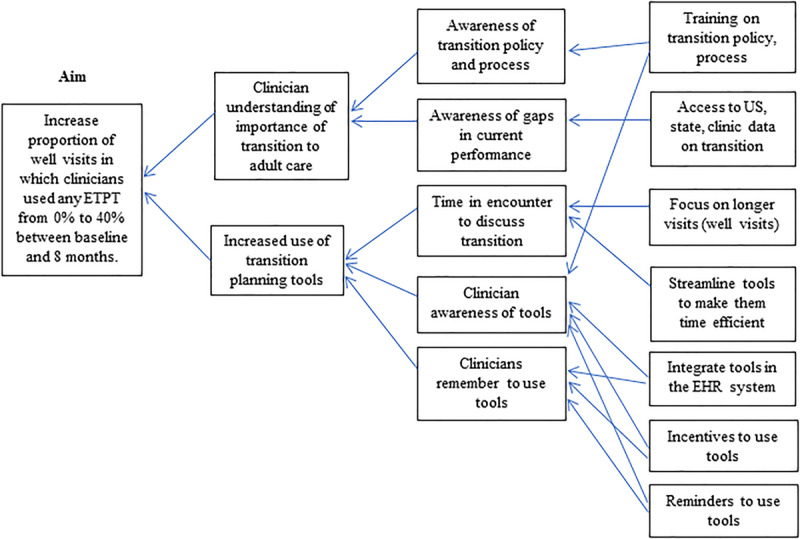
Key driver diagram. EHR indicates electronic health record; ETPT, EHR-integrated transition planning tool.

We formed a QI team consisting of an adolescent medicine physician, a nurse clinical coordinator, a pediatric resident, 3 research assistants, and an adolescent medicine fellow who served as the program leader. We conducted a needs assessment of the current transition process by reviewing policies and by soliciting input from clinicians at 2 clinic team meetings. This information was used to define the problem and key drivers (Fig. 1), to develop our theory of action, and to guide intervention development. We posited that to increase the frequency of transition discussion with youth and families, clinicians must: (1) understand the importance of transition in general, (2) have the technical skills necessary to use the tools, (3) have time in a clinical encounter to use transition planning tools, and (4) remember to use the tools. We chose specific aspects of the transition process for improvement based on the following factors: (1) inclusion of the Six Core Elements as critical components of the transition process; (2) prior work performed in our clinic; and (3) whether there were gaps in the routine use of an existing transition resource. We focused on well-visits instead of follow-up or acute visits to target visits that would include anticipatory guidance topics such as transition. We aimed to integrate transition tools into the EHR in a user-friendly and time-efficient manner based on evidence that this may improve the implementation and sustainability of transition planning efforts target.^11,14^

#### Inclusion Criteria

All completed well-visits of youth ages 17–26 during a baseline period (January 1–31, 2015) and during an intervention period (February 1 to September 30, 2015) were included. We defined “well-visits” as an office visit in which the clinician used any routine health maintenance ICD-10 code (Z00). Because the number of daily well-visits had a small range (0–11), we used the number of well-visits per week as our denominator.

#### Improvement Strategy and Interventions

**Fig. 2.**
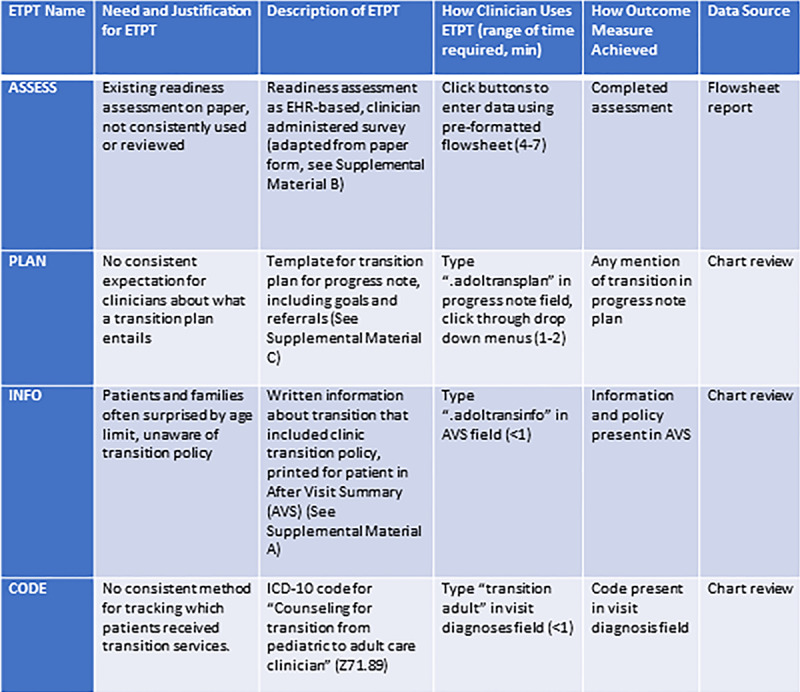
Description of EHR-integrated transition planning tools. AVS, after visit summary; EHR, electronic health record; ICD, international classification of diseases.

**Fig. 3.**
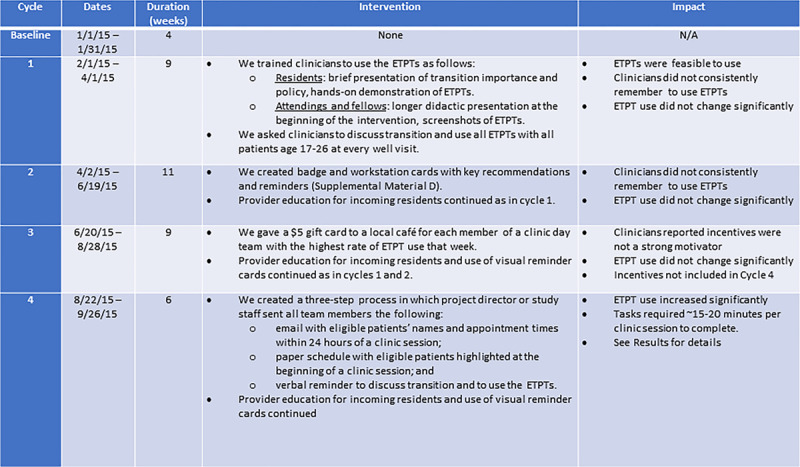
PDSA cycle interventions and impact. EHR indicates electronic health record; ETPT, EHR-integrated transition planning tool.

We developed 4 ETPTs—Assess, Plan, Info, and Code. See Figure 2 for details about ETPT development and descriptions, and supplemental materials for examples of the information brochure (**Supplemental Material A, Supplemental Digital Content 1**, http://links.lww.com/PQ9/A179), readiness assessment (**Supplemental Material B, Supplemental Digital Content 1**, http://links.lww.com/PQ9/A179), transition plan template (**Supplemental Material C, Supplemental Digital Content 1**, http://links.lww.com/PQ9/A179), and provider reminder card (**Supplemental Material D, Supplemental Digital Content 1**, http://links.lww.com/PQ9/A179). We conducted 4 PDSA cycles, with each cycle adding a layer of support for clinicians to facilitate ETPT use: (1) training, (2) visual reminders, (3) incentives, and (4) daily reminders. See Figure 3 for descriptions of the PDSA cycles. Of note, we developed the Code tool using an existing ICD10 code (Z71.89) that was chosen for tracking, not billing purposes.

### Measures

The primary outcome was “any ETPT use,” defined as the proportion of well-visits in a cycle where practitioners documented Plan, Code, or Info in the EHR. Charts were reviewed by 3 research assistants using a standardized data collection tool, and each reviewer had 2 charts verified by the project director. Plan, Code, and Info were measured differently than Assess; the former were “discrete elements” and therefore measured by chart review, whereas the latter was measured using an EHR report because it was flowsheet-based. Therefore, Assess was included as a separate outcome and was not included in “any ETPT use” because this tool was measured differently than Plan, Code, and Info, and was not linked to the other outcomes by patient. Therefore, we could not ensure that any given visit included the use of all 4 tools. In addition to analyzing “any ETPT use,” we completed a sub-analysis to examine the use of each ETPT (Plan, Code, Info, or Assess).

Clinicians provided feedback during the intervention, and adjustments were made based on this feedback and ETPT use trends, in keeping with QI methodology.^17^ We also conducted a post-intervention feedback session, which included ten clinicians (2 adolescent medicine fellows, 6 adolescent medicine faculty, 1 nurse practitioner, and 1 nurse clinical coordinator). We assessed sustainability by asking the nurse clinical coordinator and 1 physician to comment on their impressions of ETPT use over time.

### Analysis

For each outcome, we used mixed-effects logistic regression to determine if there were any differences in ETPT use between cycles. The ETPT use at baseline was zero for all outcomes—except 3 readiness assessments—therefore, we did not include the baseline data in these analyses. Comparisons of differences between all cycles were then performed with post hoc analyses, adjusting the *P* values for multiple comparisons with a Bonferroni correction.^22^ Thus, for an experiment-wise error rate of 0.05 with 6 pairwise comparisons across 4 cycles, a comparison was statistically significant if *P* < 0.008. We conducted the analyses using Stata 15.1 (STATA Corporation, College Station, TX).

ETPT use was also analyzed with a series of run charts^23^ created using Microsoft Excel (Seattle, WA) to assess the effect of the intervention on ETPT use. Data points were reported monthly because of the low frequency of well-visits by week.

### Institutional Review

The Johns Hopkins University School of Medicine Institutional Review Board determined that this project constituted QI and not human subjects research. Therefore, review and approval were not required.

## RESULTS

Input from clinicians before the intervention identified key barriers to implementing a transition process: (1) lack of awareness about national guidelines regarding transition, (2) lack of familiarity with the clinic’s existing paper-based transition resources, and (3) time constraints during clinic visits, especially with medically complex patients. All clinicians expressed a desire for training and tools to facilitate the transition process.

Most patients included in the chart review (n = 368) were female (65%) and African American (95%), with a mean age of 19.6 (SD 2.1) years, which reflects the demographics of the clinic overall.

**Fig. 4.**
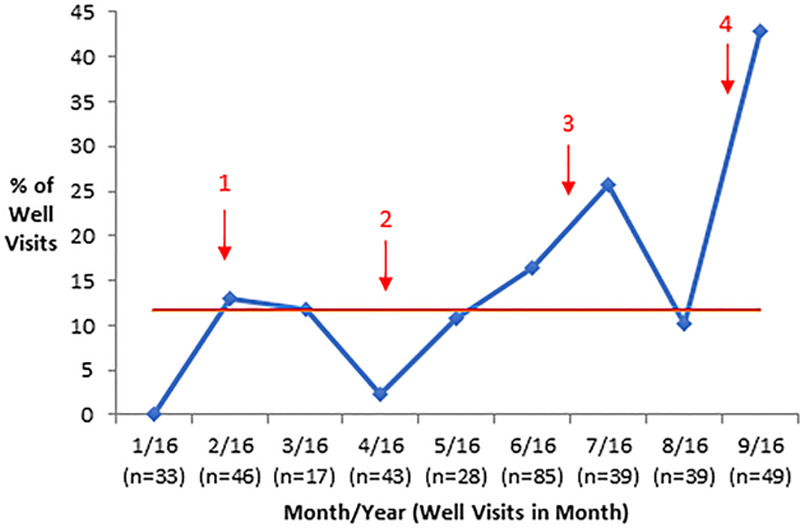
Run chart of proportion of well-visits with any EHR-integrated transition planning tool use*. ETPT indicates electronic health record transition planning tools; Red arrows, start of new PDSA cycle; Red line, median. *Any ETPT use = Plan, Info, or Code tool used during the visit. Due to differences in data sources, the assess outcome was not included. Cycle descriptions: (1) clinician training on using ETPTs, (2) visual reminders and instructions on ETPT use in the form of badge and workstation cards, (3) incentive system for clinicians who used ETPTs most often, and (4) daily, personalized reminders via email and in-person communication. See Methods section for details.

ETPT used did not change significantly in cycle 3 (incentives), so this intervention was dropped in cycle 4. Any ETPT use increased from 0% at baseline to 11.4%, 7.5%, 21.1%, and 44.9% in cycles 1–4, respectively (Table 1). The odds of any ETPT use were 10 times greater in cycle 4 compared to cycle 1 [odds ratio (OR) 10.09, 95% confidence interval (CI) 2.29–44.44, *P* = 0.002] and 22 times larger when comparing cycle 4 with cycle 2 (OR 21.99, 95% CI 3.96–122.00, *P* < 0.001) (Table 2). Run charts (Fig. 4) demonstrated an increase in any ETPT use after the start of cycles 3 and 4. However, these results reflect visual trends only because run chart rules were not applied due to few data points.^23^

**Table 1.**
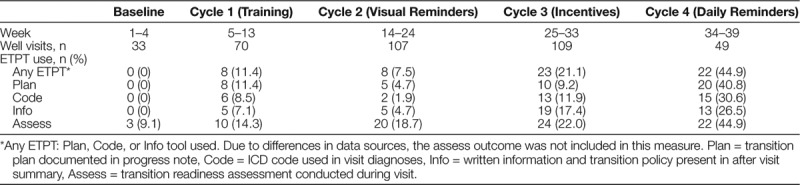
EHR-Integrated Transition Planning Tool (ETPT) Use by Cycle

**Table 2.**
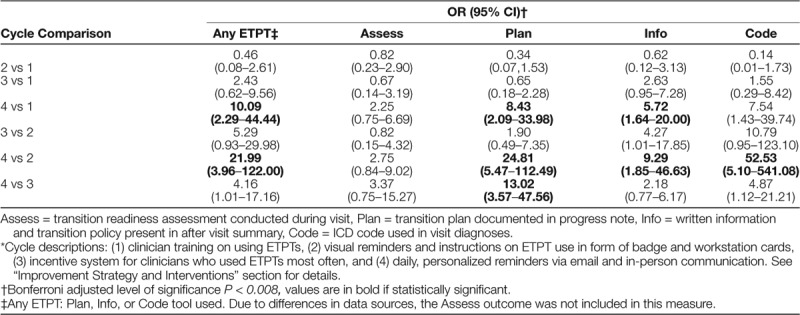
Comparisons of EHR-Integrated Transition Planning Tool (ETPT) Use by PDSA Cycle*

Regarding the sub-analysis of individual ETPTs—Plan, Code, Info, or Assess—there were statistically significant differences between cycle 4 and earlier cycles for all 4 ETPTs (Table 2). The odds of Plan use were 8 times larger in cycle 4 compared to cycle 1 (OR 8.43, 95% CI 2.09–33.98, *P* = 0.003), and 25 times larger in cycle 4 compared to cycle 2 (OR 24.81, 95% CI 5.47–112.49, *P* < 0.001). The odds of Code use were 53 times larger in cycle 4 compared to cycle 2 (OR 52.53, 95% CI 5.10–541.08, *P* < 0.001). The odds of Info use were 6 times larger in cycle 4 compared to cycle 1 (OR 5.72, 95% CI 1.64–20.00, *P* = 0.006), and 9 times larger in cycle 4 compared to cycle 2 (OR 9.29, 95% CI 1.85–46.63, *P* = 0.007). There were no statistically significant differences in the use of the Assess tool between any cycles. Run charts of individual ETPT use showed low and stable use between baseline and cycles 1 and 2, and increased use between cycles 3 and 4 (**Supplemental Materials E–H, Supplemental Digital Content 1**, http://links.lww.com/PQ9/A179).

The themes from the post-intervention feedback session were as follows. Many clinicians perceived the length of the readiness assessment to be a barrier to its implementation. Depending on a patient’s age and special health care needs, 18–27 questions were required, which took 3–10 minutes to complete. Also, clinicians identified some questions to be less relevant to their patient population, such as “Do you explain your health care needs and medical conditions to others?” At the same time, they noted other issues were missing from the assessment that may affect patients and have important implications for transition, such as unemployment or prior incarceration. Most clinicians agreed that incentives did not increase their motivation to use ETPTs, but frequent reminders were effective in keeping transition on the agenda.

Regarding sustainability, 2 years after the formal intervention, the nurse clinical coordinator reported ETPTs are continuing to be used by clinicians to assist with transition readiness assessment and planning. Anecdotal evidence suggests that fellows are using the tools most often, followed by attending physicians. In contrast, residents use them less often due to a lack of consistent training during rotation orientation and inconsistent reminders from their supervisors.

## DISCUSSION

This study suggests EHR-integrated tools such as ETPTs can help facilitate discussions about the transition to adult care during adolescent well-visits in primary care settings. We found daily, personalized reminders combined with training and visual reminders were the most effective strategy to promote tool uptake by clinicians.

To our knowledge, this is the first study to evaluate transition planning in the primary care setting using EHR-integrated tools. ETPTs appear to help fill the gap between available tools for transition planning and the EHR functionality needed to allow clinicians to efficiently and effectively use these tools in primary care.

Our findings are consistent with evidence suggesting reminders are among the most effective ways to change clinician behavior^24^ and improve quality of care.^25,26^ However, EHR-based reminders are subject to “alert fatigue”—when clinicians ignore reminders due to overuse^26^—and verbal reminders in daily huddles may be a more acceptable strategy.^27^ It may also be that the additive effects of the interventions made them more effective than each intervention alone. For example, ETPT training and visual reminders were likely necessary but not sufficient to increase ETPT use because of competing tasks during a well-visit (ie, reviewing immunization records, reconciling medications), and adding reminders allowed clinicians to more consistently incorporate these tools into their usual clinical workflow.

Clinicians cited time limitations as a major barrier to transition planning, consistent with prior studies on the challenges of addressing multiple issues in a short visit.^28^ Utilizing all members of the primary care medical home team—such as nurses, social workers, care coordinators, case managers, child-life specialists, psychologists, and parent volunteers—to manage different aspects of transition-related care may increase the efficiency of transition planning and reduce the burden on physicians for transition-related care.^5,29,30^ However, interdisciplinary interventions have not been rigorously tested or evaluated.^31,32^ Group visits are another promising model that has been used by family medicine practices for disease-specific interventions,^33^ as well as by pediatric-subspecialists, to coach youth and families through the transition to adult care.^34^ Primary care providers, likewise, could leverage ancillary staff (eg, nurse, medical assistant, registration staff, social worker) to assist with assessing and implementing transition-related tools by integrating tools into existing workflows such as during check-in or check-out periods.

The readiness assessment tool had limited uptake due to its length and lack of sociocultural relevance for our low-income, urban, African American population. Clinicians conceptualized health care transition as one part of the larger transition to adulthood, which also includes topics such as education, employment, and housing. This finding is especially salient, given that nonwhite youth receive transition services at significantly lower rates than their white peers on national surveys.^35^ Developing and implementing culturally grounded^36^ transition services—such as readiness assessments that include topics specific to local patient populations—would be likely to improve uptake from clinicians, and address the racial and ethnic disparities in the provision of transition service. Future studies should identify a small number of high impact readiness questions, as well as population-specific transition topics that may differ across clinical settings.

There are limitations to our study that we should note. In terms of the setting, because this was a single-center intervention, caution should be used in generalizing results to other settings. We did not gather data on patient, family, or provider satisfaction with the tools or the transition process. Contextual elements, including specific EHR systems, prior clinic work on transition, and institutional policies may vary across settings and likely contributed to our findings. Multi-site studies are needed to understand the impact of such contexts on future interventions better. In terms of the intervention, we did not evaluate the validity or reliability of the readiness assessment questionnaire for a predominantly African American population. Instead, the questionnaire was based on our version of a previously validated tool, which may not be culturally grounded. Additionally, the questionnaire required 7–10 minutes to administer, which may have contributed to poor uptake, especially when clinicians were seeing patients with complex medical or social needs. In terms of data and analysis, we measured “transition planning” by chart review. We defined this as any mention of transition in a progress note, which is not highly specific and may have overestimated the frequency of planning. Future studies are needed to determine which variables best measure successful transition, how to elicit these variables efficiently, and how to track them within an EHR system. Cycle comparisons should be interpreted with the understanding that interventions were added from one cycle to the next, except for incentives, which we stopped at the end of cycle 3. While this approach is consistent with QI methodology,^17^ it makes direct comparisons between cycles more challenging. CIs were wide in certain comparisons, especially when including data from cycle 2 because a small absolute number of ETPTs were used by clinicians. Due to the limited numbers of well-visits per week, we used monthly data for the run charts, which did not provide enough data points to allow us to apply run chart rules using accepted guidelines.^23^ Run chart rules can be applied to situations with few data points.^23^ However, we chose to take a more conservative approach and included charts only as a visual display of outcome data to inform a future investigation. Nonetheless, our findings suggest ETPTs may be a key component of improving transition-related care.

## CONCLUSIONS

EHR-integrated tools to facilitate the transition from pediatric to adult health care are feasible to implement. Daily personalized reminders combined with training and visual reminders, were the most effective methods for increasing tool use. Transition readiness assessments may be more efficient and impactful when brief, socioculturally-relevant, and applicable to broader aspects of the transition to adulthood beyond health care. Developers and policy-makers will need to consider clinician factors such as time availability and alert fatigue in the process of creating, implementing, and evaluating future interventions.

## ACKNOWLEDGMENTS

We thank all the youth, families, and clinicians who contributed their time and efforts to this study. Statistical support was provided by Carol B. Thompson at the Johns Hopkins Biostatistics Center and supported by a grant from the National Center for Research Resources and the National Center for Advancing Translational Sciences (NCATS) of the National Institutes of Health (grant number 1UL1TR001079). We also acknowledge Patricia Flanagan and Constance Baldwin for their valuable comments on early drafts of this manuscript. We also acknowledge the staff of Parents Place of Maryland for their support of this project and their ongoing advocacy for youth and families. This work was supported by the National Institutes of Health, Adolescent Health Promotion Research Training Program (T32HD052459-07, PI: Maria Trent); Maternal and Child Health Bureau, Leadership Education in Adolescent Health (T71MC08054, PI: Hoover Adger); Maryland Department of Health and Mental Hygiene (90064008, PI: Renata Sanders); and Parents Place of Maryland (120582, PI: Renata Sanders).

## DISCLOSURE

The authors have no financial interest to declare in relation to the content of this article.

## Supplementary Material


