# Supplementary material for: Improvement of Electronic Health Record Integrated Transition Planning Tools in Primary Care
Source: Pediatr Qual Saf. 2020 May 18;5(3):e282. doi: 10.1097/pq9.0000000000000282 (PMC7297398; doi:10.1097/pq9.0000000000000282)
Supplement: Supplementary file 1 [file pqs-5-e282-s001.docx]

**Preparing for the Future: Transition from Pediatric to Adult Health Care**

Young people must stay healthy in order to find success in the classroom, in the community, and on the job. Taking charge of one’s health is part of growing up, becoming independent, and finding one’s place in the adult world.

**Harriet Lane Transition Policy Statement**

We are committed to helping all of our patients make a smooth transition from pediatric to adult health care by 26 years old. This process requires working with patients and families to plan and prepare for transition starting around the 17^th^ birthday. At age 18, youth in our practice will transition to an adult model of care with modifications as needed for youth with special health care needs. The goal of transitioning to the adult model of care is to provide youth with the skills necessary to independently access health care services as an adult in a confidential and protected manner.

# What is Health Care Transition?

Health care transition involves helping teens and young adults move from child-based care to adult care. This process takes time and includes:

- Encouraging independence starting at a young age, to get ready for adult life
- Shifting responsibilities from the parent to the young person
- Helping teens and young adults make their own health care decisions
- Choosing an adult health care provider and transferring care

*Celebrate transition as an important milestone in a young person’s life!*

# 10 Steps Toward Successful Transition

***1. Start early.*** Beginning at age 14, write down future goals and how to achieve them. Learn about health, how to care for oneself, and how to make appointments.

***2. Learn how adult care is different from pediatrics.*** Understand that adult providers want patients to discuss issues with them in an honest and clear way. They want patients to follow through on plans and make their own decisions.

***3. Develop a medical summary.***  This is a one-page form carried in the wallet. It includes the patient’s medicines and allergies. Medical problems and hospital stays are also listed. It is useful for emergencies or when applying for benefits. Take it when visiting new providers. If there are many medical needs, a care notebook may also be helpful (see [www.hrtw.org](http://www.hrtw.org)).

***4. Know your health insurance options.*** Learn what types of plans youth may obtain as an adult. Primary Adult Care (PAC) is available for those age 19 and over with limited income. Private insurance from work is another option. If parents have private coverage, they can insure their children up to age 26. Medicaid and Supplemental Security Income (SSI) rules change after age 18. Be sure to learn about these differences.

PAC: 1-800-226-2142 [http://www.dhmh.state.md.us/mma/ pac/index.htm](http://www.dhmh.state.md.us/mma/%20pac/index.htm)

Medical Assistance: 1-800-735-2258 [http://www.dhmh.state.md.us/ ma4families/index.html](http://www.dhmh.state.md.us/%20ma4families/index.html)

Priority Partners: 1-800-654-9728 [www.ppmco.org](http://www.ppmco.org)

***5. Understand what age 18 means in legal terms.*** Some young people cannot make adult health care decisions due to illness or disability. In these cases, another adult will need to apply for legal care of the youth once he turns 18 years old.

***6. Practice health skills.*** Eat well and exercise. Make healthy choices. Avoid drugs, alcohol, and tobacco. Talk with providers and family about sex and relationships. Encourage teens to make their own appointments and prescription refills.

***7. Find and use adult care.*** Gather names of adult primary care providers and specialists. Ask your current provider, insurance company, friends, and support groups for help.

***8. Learn how to communicate with providers.*** Be open and honest. Bring the medical summary to heath visits. Know about medicines and any allergies. Bring a friend for help remembering information. Ask your provider for materials to take home. Call the clinic later with any questions or problems.

***9. Seek support from schools and other agencies.* Ask your provider about Health Leads, which helps connect youth and family with a wide variety of resources, and is located within our clinic.** Health Leads, as well as our team can help you with:

- Getting ready for college
- Career guidance
- GED programs
- Job training
- Living on your own
- Disability resources
- Health support systems

***10. Use community resources if youth have special needs.*** Learn about transition and resources at these websites:

Maryland Division of Rehabilitation Services: [www.dors.state.md.us](http://www.dors.state.md.us)

Family NETworks: [www.family-networks.org/teen.cfm](http://www.family-networks.org/teen.cfm)

Healthy and Ready to Work National Center: [www.hrtw.org](http://www.hrtw.org)

National Health Care Transition Center: [www.gottransition.org](http://www.gottransition.org)

Kennedy Krieger Institute resource finder: [www.resourcefinder.kennedykrieger.org](http://www.resourcefinder.kennedykrieger.org)

**Contact us if you have any questions:**

Harriet Lane Primary Care

Rubenstein Building

200 North Wolfe Street

Baltimore, MD 21287

410-955-5710
