# Supplementary material for: Improvement of Electronic Health Record Integrated Transition Planning Tools in Primary Care
Source: Pediatr Qual Saf. 2020 May 18;5(3):e282. doi: 10.1097/pq9.0000000000000282 (PMC7297398; doi:10.1097/pq9.0000000000000282)
Supplement: Supplementary file 2 [file pqs-5-e282-s002.docx]

***Supplemental Materials* *B: Transition readiness assessment.*** This questionnaire was originally adapted from the TRAQ by adding questions about specific transfer events (i.e. made appointment with adult physician). We integrated it into our EHR system so it could be administered by a clinician and tracked easily over time.

**Transition Readiness Assessment**

**Person completing survey: ___ Adolescent ___ Parent/Guardian**

**Instructions: Please complete these questions based on how you feel about yourself (or your adolescent)**

| ***I. Managing Your Daily Activities*** | **No, I do not know how**  **(1)** | **No, but I want to learn**  **(2)** | **Yes, I am learning to do this**  **(3)** | **Yes, I have started doing this**  **(4)** | **Yes, I always do this**  **(5)** |
| --- | --- | --- | --- | --- | --- |
| 1. Do you help plan or prepare meals/food? |  |  |  |  |  |
| 2. Do you manage your money & budget household expenses (for example: using credit/debit card)? |  |  |  |  |  |
| 3. Do you keep a calendar or list of your appointments and activities? |  |  |  |  |  |
| 4. Do you carry a state ID or driver’s license with you every day? |  |  |  |  |  |
| **DOMAIN I TOTAL (out of 20)** |  |  |  |  |  |

| ***II. Managing Your Health*** | **No, I do not know how**  **(1)** | **No, but I want to learn**  **(2)** | **Yes, I am learning to do this**  **(3)** | **Yes, I have started doing this**  **(4)** | **Yes, I always do this**  **(5)** |
| --- | --- | --- | --- | --- | --- |
| 1. Do you explain your health care needs and medical conditions to others? |  |  |  |  |  |
| 2. Do you answer questions that are asked by the doctor, nurse, or clinic staff? |  |  |  |  |  |
| 3. Do you fill a prescription if you need to? |  |  |  |  |  |
| 4. Do you take medications correctly and on your own? |  |  |  |  |  |
| **DOMAIN II TOTAL (out of 20)** |  |  |  |  |  |

| ***III. Taking Charge of Your Health Care*** | **No, I do not know how**  **(1)** | **No, but I want to learn**  **(2)** | **Yes, I am learning to do this**  **(3)** | **Yes, I have started doing this**  **(4)** | **Yes, I always do this**  **(5)** |
| --- | --- | --- | --- | --- | --- |
| 1. Do you call the doctor’s office to make an appointment? |  |  |  |  |  |
| 2. Do you make a list of questions before your doctor’s visit? |  |  |  |  |  |
| 3. Do you arrange for your ride to medical appointments? |  |  |  |  |  |
| 4. Do you fill out the medical history form, including a list of your allergies? |  |  |  |  |  |
| **DOMAIN III TOTAL (out of 20)** |  |  |  |  |  |

| ***IV. Being Prepared*** | **No, I do not know**  **(1)** | **No, but I want to learn**  **(2)** | **Yes, I am learning about this**  **(3)** | **Yes, I usually know**  **(4)** | **Yes, I always know**  **(5)** |
| --- | --- | --- | --- | --- | --- |
| 1. Do you know what to do in case you have an emergency? |  |  |  |  |  |
| 2. Do you know when you need to contact your doctor (for example: you are sick or you need a prescription)? |  |  |  |  |  |
| 3. Do you know what your health insurance covers? |  |  |  |  |  |
| 4. Do you know how to apply for health insurance if you lose your coverage? |  |  |  |  |  |
| **DOMAIN IV TOTAL (out of 20)** |  |  |  |  |  |

**Are you 17-years-old or older? YES NO**

*If you answer “YES”, please complete part V. Transition to Adult Health Care.*

| ***V. Transition to Adult Health Care*** | **No, I do not know how**  **(1)** | **No, but I want to learn**  **(2)** | **Yes, I am learning to do this**  **(3)** | **Yes, I have started doing this**  **(4)** | **Yes, I have done this**  **(5)** |
| --- | --- | --- | --- | --- | --- |
| 1. Have you found a clinic or provider where you will go for adult medical care? |  |  |  |  |  |
| 2. Have you made an appointment with your new medical provider? |  |  |  |  |  |
| 3. Have you created a medical summary with your doctor? |  |  |  |  |  |
| 4. Have you transferred your medical records and obtained a copy of your immunizations? |  |  |  |  |  |
| **DOMAIN V TOTAL (out of 20)** |  |  |  |  |  |

**Do you see a specialist for any health problems? YES NO**

(NOTE: This includes any medical or mental health specialist)

*If you answer “YES”, please complete part VI. Preparing for the Future.*

**Do you receive any special educations services? YES NO**

*If you answer “YES”, please complete part VI. Preparing for the Future.*

NOTE: Please answer these questions if they apply to you and your family.

| ***VI. Preparing for the Future*** | **No, I do not know how**  **(1)** | **No, but I want to learn**  **(2)** | **Yes, I am learning to do this**  **(3)** | **Yes, I have started doing this**  **(4)** | **Yes, I have done this**  **(5)** | **N/A** |
| --- | --- | --- | --- | --- | --- | --- |
| 1. Have you learned to recognize your symptoms that need quick medical attention? |  |  |  |  |  |  |
| 2. Do you help with routine maintenance of your medical equipment so that it is in good working condition? |  |  |  |  |  |  |
| 3. Have you applied for supplemental security insurance (SSI)? |  |  |  |  |  |  |
| 4. Have you obtained additional help with school/community resources or assisted living? |  |  |  |  |  |  |
| 5. Have you and your family developed a legal Power of Attorney for health care decisions in the event that your health changes and you are unable to make decisions for yourself? |  |  |  |  |  |  |

*Adapted from Transition Readiness Assessment Questionnaire 5.0, © Wood, Sawicki, Reiss &, Livingood 2012
