# Supplementary material for: Improvement of Electronic Health Record Integrated Transition Planning Tools in Primary Care
Source: Pediatr Qual Saf. 2020 May 18;5(3):e282. doi: 10.1097/pq9.0000000000000282 (PMC7297398; doi:10.1097/pq9.0000000000000282)
Supplement: Supplementary file 3 [file pqs-5-e282-s003.docx]

***Supplemental Materials* *C: Transition plan.*** We developed a template that clinicians could use to document the content of their transition-related discussions with patients and families. We asked clinicians to include this in their progress note, as well as the AVS.

**Transition to Adult care**

- Readiness assessment conducted: yes
- We discussed transition today. Specifically, we talked about our clinic policy to transfer to adult care prior to your 26^th^ birthday, and the importance of transition.
- The following written resources were provided: policy and brochure
- You agreed to work on to following skills: Making your own appointments and calling the pharmacy to request refills on your medications
- At this time, you have taken the following steps toward a successful transition: Determined work and/or school plan, determined housing situation for the future
- Referrals made: Health Leads to discuss employment
- Please make an appointment to return to clinic in 2 months
