# Supplementary material for: Improvement of Electronic Health Record Integrated Transition Planning Tools in Primary Care
Source: Pediatr Qual Saf. 2020 May 18;5(3):e282. doi: 10.1097/pq9.0000000000000282 (PMC7297398; doi:10.1097/pq9.0000000000000282)
Supplement: Supplementary file 4 [file pqs-5-e282-s004.docx]

Adolescent         Transition Process y

1) Discuss transition with patient 4) Document in Visit Diagnosis s

• Age to start transition discussion: 17                - Under Visit Navigator

• Age to initiate transfer process: 23 (see back)   • Click Visit Diagnosis

2) Give readiness assessment      • Type in Text Box “Transition Adult”

- Under Visit Navigator Tab                                 • Click “Add” then Click “Accept”→

• Click on Disease Management                       5) Put information in AVS (Patient Instructions)

• Click on “Adol Trans Readiness”                      - Plan: Dotphrase “.adoltransitionplan”

3) Refer patient depending on readiness needs - Brochure: Dotphrase “.adoltransitionbrochure”

- See Referral Guide in preceptor room                 6) Document in Progress Note

• Health Leads: Place EPIC referral               - Use dotphrase “.adoltransitionplan”

   • Social Work: Place EPIC referral or PING SW - Shortcut: cut and paste from AVS!

   • Kathy: PING if urgent, email if non-urgent

Every Youth age 23 and older, EVERY visit t Adolescent Transition Team Directory y

1) Assess Providers r

• Conduct readiness assessment Jack Rusley [***@jhmi.edu](mailto:***@jhmi.edu)

• Provide counseling as needed Renata Sanders [***@jhmi.edu](mailto:***@jhmi.edu)

• Refer & provide information (see front) Kathy Tomaszewski [***@jhmi.edu](mailto:***@jhmi.edu)

2) Remind Research Assistants s

• Youth that they must transfer at age 25 Larnce Robinson [***@jhmi.edu](mailto:***@jhmi.edu)

3) Ask Rana Saeed [***@jhu.edu](mailto:***@jhu.edu)

• Have you started the transfer process?

4) Refer and follow up r

• Email transition team for support

• Schedule follow up visit to discuss transition
